# Heterochronous mitogenomes shed light on the Holocene history of the Scandinavian brown bear

**DOI:** 10.1038/s41598-024-75028-6

**Published:** 2024-10-22

**Authors:** Isabelle Sofie Feinauer, Edana Lord, Johanna von Seth, Georgios Xenikoudakis, Erik Ersmark, Love Dalén, Ioana-Nicoleta Meleg

**Affiliations:** 1 Centre for Palaeogenetics, Svante Arrhenius väg 20C, 106 91 Stockholm, Sweden; 2 Department of Zoology, Stockholm University, Svante Arrhenius väg 18C, 106 91 Stockholm, Sweden; 3 Department of Bioinformatics and Genetics, Swedish Museum of Natural History, Box 50007, 104 05 Stockholm, Sweden; 4 Department of Archaeology and Ancient Culture, Wallenberglaboratoriet, Lilla Frescativägen 7, Stockholm University, 106 91 Stockholm, Sweden; 5 Emil G. Racoviță Institute, Babeș-Bolyai University, Clinicilor 5-7, 400006 Cluj-Napoca, Romania; 6 Emil Racoviță Institute of Speleology of the Romanian Academy, Calea 13 Septembrie 13, 050711 Bucharest, Romania

**Keywords:** Brown bear, Mitogenomes, Ancient DNA, Scandinavia, Postglacial recolonisation, Bottleneck, Evolutionary genetics, Phylogenetics

## Abstract

Following glacial retreat after the last ice age, brown bears (*Ursus arctos*) recolonised Scandinavia. Previous research based on mitochondrial markers suggests that bears recolonised from both the north and the south, with a contact zone in central Scandinavia. More recently, the Scandinavian brown bear was subjected to a strong population decline with only ca. 130 remaining individuals, due to intense human persecution approximately 100 years ago. Here, we analyse 41 ancient, historical, and modern mitochondrial genomes, to examine the number of female lineages involved in the postglacial recolonisation event and temporal changes in the Scandinavian brown bears’ mitochondrial genetic diversity. Our results support the bi-directional recolonisation hypothesis, indicating multiple mitochondrial lineages from clade 1a possibly followed a southern route, while only a single lineage from clade 3a appears to have followed a northern route. Furthermore, we found that the recent bottleneck had a strong impact on the southern subpopulation, resulting in only one remaining haplotype in the contemporary brown bears. For the northern subpopulation, the impact was moderate, and most haplotypes were retained throughout the bottleneck. By exploring the postglacial recolonisation and recent population pressures, our study enhances understanding of how these factors have influenced the genetic diversity of Scandinavian brown bears.

**Supplementary Information:**

The online version contains supplementary material available at 10.1038/s41598-024-75028-6.

## Introduction

The flora and fauna of Europe have been profoundly shaped by the last ice age and the following warming climate of the Holocene^1^. Population contraction into glacial refugia during periods of harsh climate conditions was key for the survival of temperate species^1–3^. Glacial refugia were ice-free areas, mostly found in southern Europe, e.g. Iberia, Italy, the Balkans, and the Caucasus^4,5^. However, northern refugia have also been described^6^, such as in the Carpathians^7^, the Ural mountains^8^, and Andøya in Norway^9^. The potential genetic consequences of isolation in refugia have been described in the expansion contraction model^2^. It states that the isolation in refugia during harsh climate conditions leads to genetic differentiation, while during periods with favourable climate, species expand their ranges, resulting in possible overlap and admixture. At the end of the last ice age, temperate species expanded from isolated refugia into Europe. This postglacial recolonisation has been proposed to be one of the main drivers of the phylogeographic patterns found today^1,2^.

The Last Glacial Maximum (LGM) was reached between 26.5 and 19 kya (thousand years ago)^10^, during which northern Europe was covered by the Fennoscandian ice sheet^11^. After reaching its maximum span around 21 kya, the Fennoscandian ice sheet started to retreat^11^, which opened the landscape for a recolonising flora and fauna. The deglaciation of Scandinavia started around 16 to 15 kya and Scandinavia became virtually ice free ca. 9.7 kya, when the last remains of the ice sheet in Sarek, Sweden melted^12^. There are four main hypothesis regarding the postglacial recolonisation of Scandinavia^2^: (1) A northern route through Finland, observed in species like arctic foxes^13^, red foxes^14^, and wood lemmings^15^; (2) A southern route via land bridges to Sweden, known to be taken by European hedgehogs^16^; (3) Recolonisation from both directions, seen in common toads^17^, shrews^18^, humans^19^, and field voles^20^; (4) Expansion from local refugia within Scandinavia, as hypothesised for pine and spruce^9,21^, and Norwegian lemmings^22^.

In the context of phylogeography and conservation genetics, the European brown bear (*Ursus arctos*) represents a key study species. Its current phylogeographic patterns offer valuable insights into the impact and consequences of past climatic changes and human actions^23–26^. For many years, brown bears have been considered in a phylogeographic context as a temperate species that primarily survived the last glacial period in the established southern refugia^1,3^. The results of early phylogeographic studies based on short mitochondrial sequences suggested a postglacial recolonisation of Europe from refugia in Iberia, Italy and the Balkans, and the Caucasus or Carpathian mountains, of bears belonging to mitochondrial western clades 1a, 1b, and eastern clade 3a respectively^24,27–29^. However, Ersmark et al.^30^ revealed a more complex history of European brown bears during the last ice age. Their findings suggested that brown bears belonging to mitochondrial clade 1a not only survived in the established southern refugia, but also persisted in less commonly recognised mid-latitude European locations throughout the LGM, including areas such as northern France and Belgium.

Previous studies have suggested that brown bears recolonised Scandinavia from two directions^1,24,27^. Based on phylogeographic studies using short mitochondrial sequences, it has been proposed that brown bears from the Western European refugia recolonised Scandinavia through the southern route over the opening land bridges, while brown bears from the Eastern European refugia expanded into northern Scandinavia via Finland^24,25,30–32^. The bi-directional recolonisation resulted in the presence of two distinct haplogroups, the western (1a) in the South and the eastern (3a) in the North of Scandinavia. Today, the two haplogroups meet within a contact zone in central Scandinavia^33^, which has been proposed to be a remnant of the bi-directional postglacial recolonisation and likely was retained due to female philopatry in brown bears^32^. However, Ersmark et al.^30^ reported early Holocene remains of brown bears in southern Scandinavia carrying a clade 3 haplotype, which could indicate that Eastern clade 3a brown bears might have recolonised Scandinavia through both routes, and that recent processes may have further affected the present-day genetic structure and diversity.

In the early 20th century, the Scandinavian brown bear experienced a dramatic population decline due to intense human persecution^34,35^. In the mid 1800’s the brown bear population size was ca. 1650 individuals in Sweden and ca. 3100 individuals in Norway, dropping to 130 surviving individuals in Sweden and a virtual extinction in Norway by the 1930’s^35^. To rescue the species from extinction, protective actions were taken around the early 20th century by the Swedish and later the Norwegian governments^34,35^, which success was reflected in a subsequent population recovery^35–37^, with an estimate of around 2900 brown bears flourishing in the forests of Sweden by 2017^38^. Today, the brown bear’s distribution in Scandinavia is limited to the northern and central regions, as well as a limited area south of, but close to, the contact zone in Jämtland, Sweden^34,38^. The mitochondrial diversity of Scandinavian brown bears in the Holocene, and to which extent it has been impacted by the intense population decline, has been addressed in previous studies based on short mitochondrial markers^32,39^. Bray et al.^32^ attributed low mitochondrial diversity primarily to the postglacial recolonisation and founder effects, and Xenikoudakis et al.^39^ emphasised the impact of the recent bottleneck on the mitochondrial diversity loss.

In this study, we present an analysis of 41 mitochondrial genomes of Scandinavian brown bears. We aimed to further elucidate the postglacial recolonisation of Scandinavia, with a focus on achieving a clearer phylogenetic resolution. Moreover, we investigated how recent human impacts, leading to near extinction, have influenced the lineage structure and diversity of Scandinavian brown bears based on mitochondrial data.

## Results

### Dataset

**Fig. 1:**
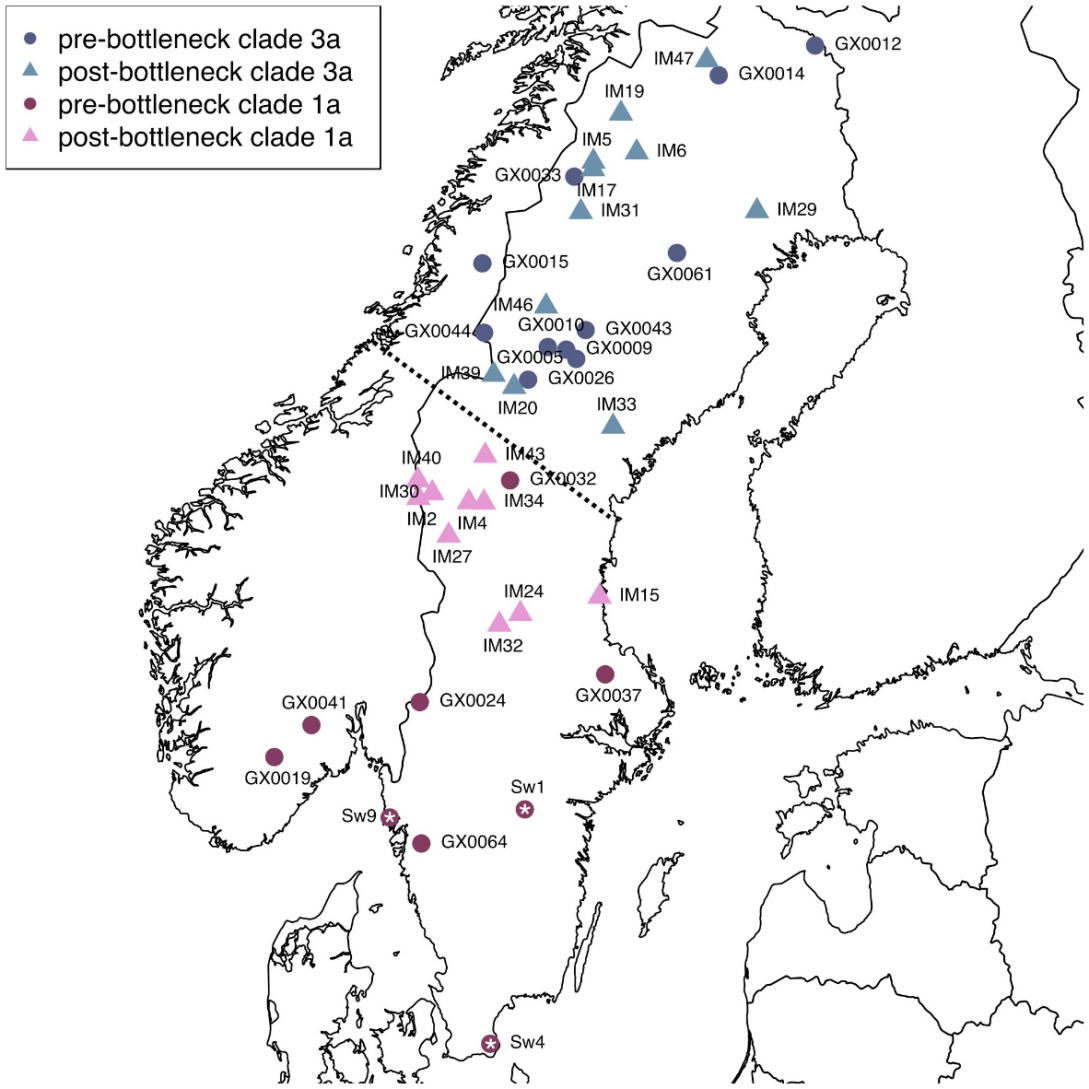
The sampling locations of the 41 brown bears (*Ursus arctos*). Brown bears carrying mitochondrial clade 1a are displayed in red and carrying clade 3a are displayed in blue. Symbols indicate the time period, with dark-coloured circles representing the three ancient samples (early Holocene; marked with star) and the 17 historical pre-bottleneck samples (1830 to before 1953), and light-coloured triangles representing the modern post-bottleneck samples (2007–2012). The dotted line represents the approximate geographic location of the contact zone of the two mitochondrial clades. The map was generated using the R package rworldmap^40^.

The study focuses on Scandinavian brown bear samples (*Ursus arctos*) including three ancient specimens dated between 10,290 and 1,916 cal years BP, 17 historical samples from the 1830s to before 1953, and 21 modern samples collected from 2007 to 2012 (Fig. 1). We generated 41 mitogenomes with a minimum average coverage of 5X. The 21 modern samples yielded an endogenous DNA content above 99%, the 17 historical samples had a varying endogenous DNA content between 3 and 90%, and the endogenous DNA content of the three ancient samples was between 0.4 and 11% (Supplementary Tables S2, S3).

### Phylogenetic relationship and divergence

**Fig. 2:**
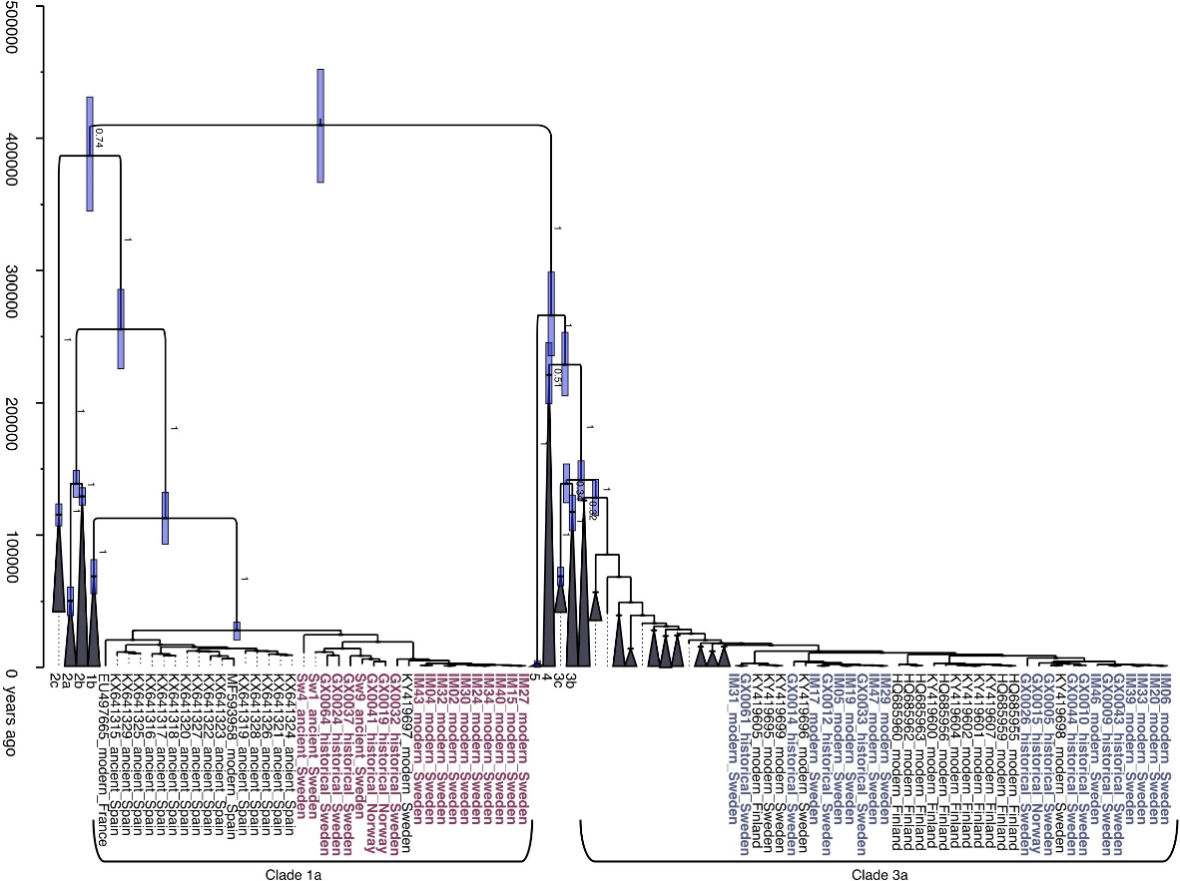
Time-calibrated phylogeny of 449 brown and polar bear mitogenomes (15,118 bp) generated in BEAST v1.10.4, using a substitution rate of 2.48 × 10^− 8^ substitutions per site per year. The y-axis is given in years ago. Individuals are labelled with SampleID or Genbank Accession Number, time period, and country. Collapsed clades are labelled by number. Samples from Scandinavian brown bears sequenced in this study are shown in red (clade 1a) and blue (clade 3a). Branch posterior probability until clade level is displayed, and bars indicate the 95% highest posterior density (HPD) for nodes.

We constructed a Bayesian phylogenetic tree of 449 brown bear and polar bear mitogenomes, consisting of the 41 Scandinavian brown bear samples generated in this study and 408 previously published samples (Fig. 2). The mitochondrial clades 1–5 were represented, with the 26 polar bears forming clade 2b. The Bayesian phylogeny supported the presence of brown bears from two distinct mitochondrial clades in Sweden, clade 1a and clade 3a. All Scandinavian brown bears originating from south of the contact zone fall into clade 1a, while all brown bears located north of the contact zone were found within clade 3a. This pattern was consistent for ancient, historical, and modern samples.

**Fig. 3:**
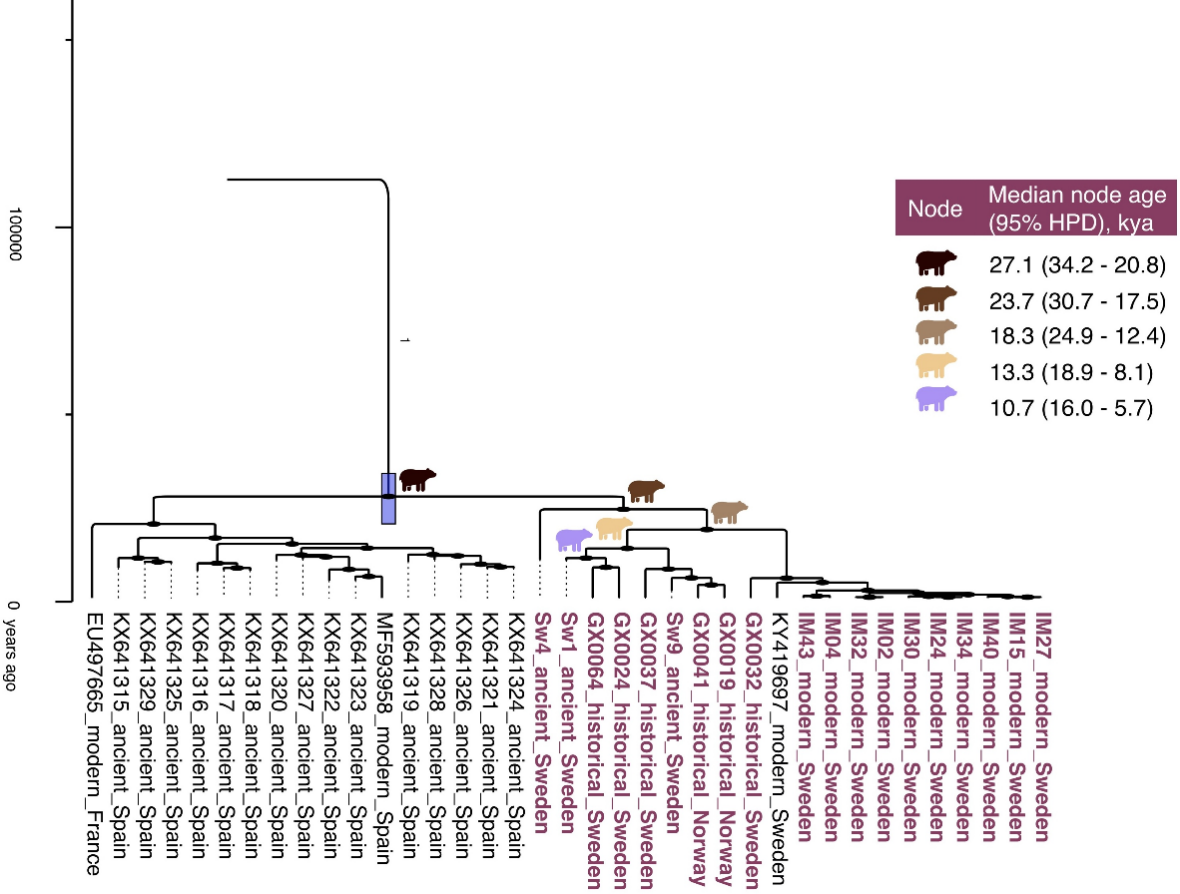
Time-calibrated phylogeny of clade 1a, zoomed in from Fig. 2. The y-axis is given in years ago. Samples from this study are displayed in red. Labelling follows Genbank Accession number or SampleID, timeframe categories, and country. Posterior probability and the 95% HPD of the oldest node is displayed. For the five oldest nodes, within the Scandinavian brown bears, marked with bear symbols, the median node age and 95% HPD in kya are listed in the table.

The Scandinavian brown bears assigned to clade 1a clustered together with brown bears from France and Spain, which were previously identified as clade 1^41,42^ (Figs. 2 and 3). The estimated divergence time of the Scandinavian brown bear lineage from its Spanish and French relatives was estimated to 27.1 kya (95% HPD: 34.2–20.8 kya). The time of the most recent common ancestor of all southern Scandinavian brown bears was estimated to 23.7 kya (95% HPD: 30.7–17.5 kya), when the oldest sample (Sw4; 10290 ± 57 calibrated years before present) diverged from the remaining samples. All post-bottleneck southern Scandinavian brown bears cluster together, with a most recent common ancestor estimated to 4.1 kya (95% HPD: 7.3–1.9 kya).

**Fig. 4:**
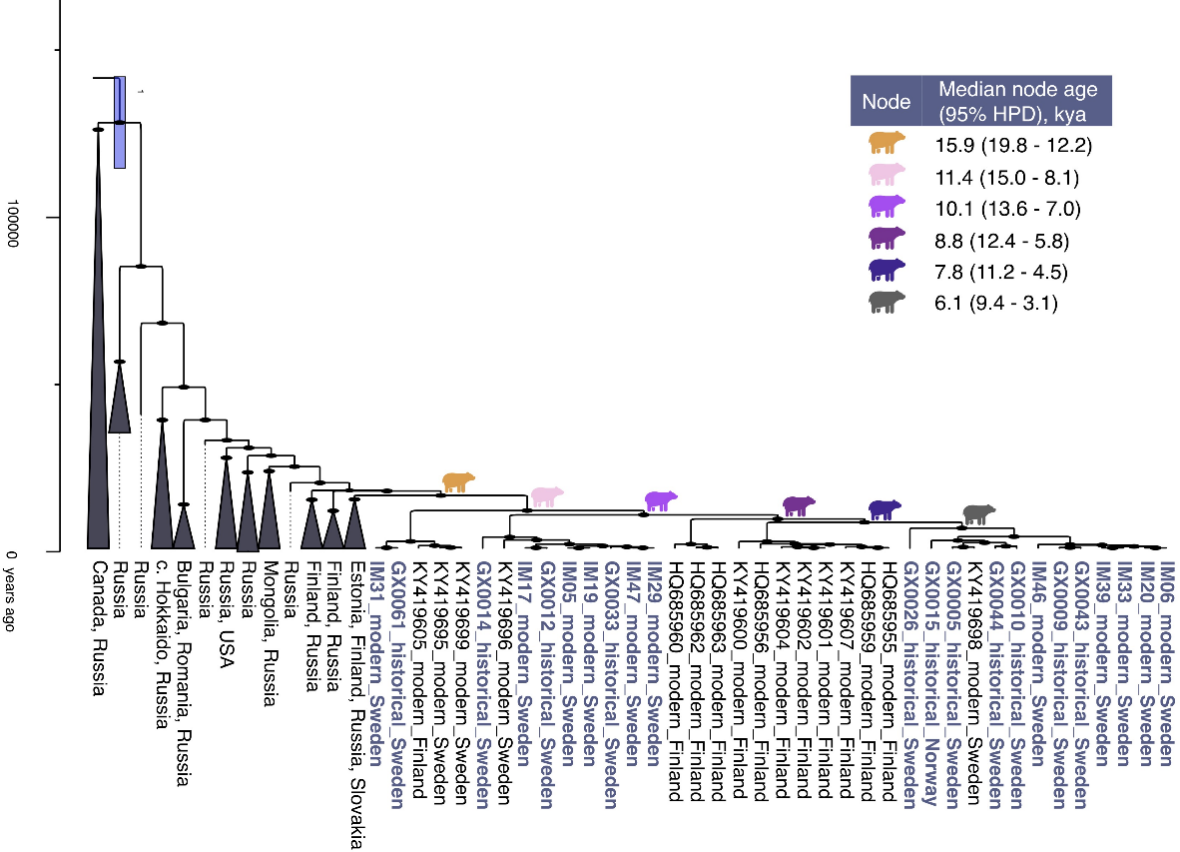
Time-calibrated phylogeny of clade 3a, zoomed in from Fig. 2. New samples presented in this study are displayed in blue. Labels correspond to SampleID or Genbank accession number, time period, and country, for individual samples and collapsed branches respectively. The node posterior probability is given together with the node branch as 95% HPD for the node forming the clade. The median node ages with 95% HPD in kya of the six oldest nodes within the Scandinavian bears are given in the table, corresponding to the coloured bear symbols. The y-axis is labelled in years ago.

Clade 3a comprised the northern Scandinavian brown bears and previously published brown bears from Finland, Estonia, Russia, Romania, Bulgaria, Slovakia, Mongolia, USA, Canada, and central Hokkaido (Figs. 2 and 4). The northern Scandinavian brown bears from Norway and Sweden are nested within a clade comprising some brown bears from Finland as well. The estimated divergence time between this group and the remaining clade 3a bears was estimated to 15.9 kya (95% HPD: 19.8–12.2 kya). The most recent common ancestor for this joint Scandinavian and Finnish group was estimated to 11.4 kya (95% HPD: 15.0–8.1 kya).

### Mitogenome haplotypes and diversity

**Fig. 5:**
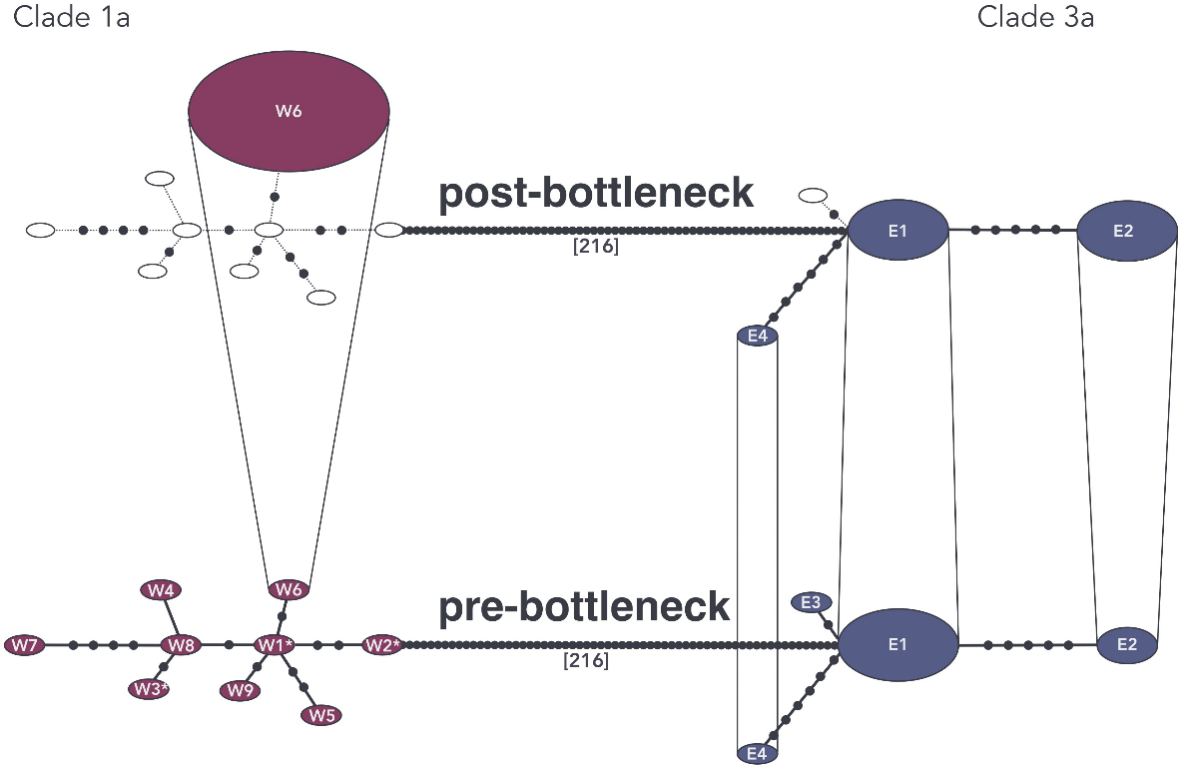
Temporal haplotype network of the 41 brown bear mitogenomes (11,412 bp). Red indicates the southern Scandinavian samples, carrying the western haplogroup belonging to clade 1a. Blue represents the northern Scandinavian brown bears, carrying the eastern haplogroup belonging to clade 3a. The number of mutations that separate the two haplogroups are given in square brackets. The network is separated by time, before (bottom) and after the bottleneck (top). Coloured circles display individual haplotypes, labelled with haplotype ID, and are connected by solid lines. Empty circles represent haplotypes that are not found in the corresponding time period and are connected by dashed lines. Black dots on the lines represent missing haplotypes. Haplotypes that are continuous throughout the two time periods are connected by vertical lines. The size of the ellipse indicates the number of individuals assigned to a haplotype. An asterisk next to the haplotype ID indicates the ancient samples.

Consistent with previous studies, we found two distinct haplogroups within the 41 Scandinavian brown bear mitogenomes. All samples from southern Scandinavia fall into the western haplogroup of clade 1a, while all samples from northern Scandinavia carry the eastern haplogroup of clade 3a. Before the bottleneck, a total of 13 haplotypes in 20 brown bears was observed. In the 21 post-bottleneck brown bears, only four haplotypes were found (Fig. 5; Supplementary Figure S2).

For the southern Scandinavian brown bears, nine haplotypes among nine pre-bottleneck samples and one haplotype among ten post-bottleneck samples were present. All post-bottleneck southern samples share a haplotype with the pre-bottleneck sample GX0032, from Jämtland. The three ancient southern samples, Sw1, Sw4, and Sw9, carry the haplotypes W1, W2, and W3 respectively, with Sw1 and Sw4 being separated by three and Sw1 and Sw9 being separated by four segregating sites, respectively.

Among the brown bears from northern Scandinavia, the eleven pre-bottleneck samples showed four different haplotypes. Three of those haplotypes were retained in the eleven post-bottleneck northern samples. The majority (19 out of 22) of the northern samples were assigned to haplotype E1 and E2, with six pre- and five post-bottleneck, and three pre- and five post-bottleneck samples, respectively. Haplotype E3 was found only in a single sample, GX0026, and not present in the post-bottleneck data, while haplotype E4 was represented by one sample before and after the bottleneck, GX0061 and IM31.

We observed the highest haplotype and nucleotide diversity among southern Scandinavian brown bears before the bottleneck, and the lowest among the southern Scandinavian brown bears after the bottleneck (Table 1). Haplotype and nucleotide diversity in the post-bottleneck southern samples was zero, as all samples fell into the same haplotype. For the northern Scandinavian brown bears, haplotype and nucleotide diversity were similar before and after the bottleneck. Tajima’s D was not significant in any of the datasets. Due to the lack of segregating sites, Tajima’s D was not estimated for the post-bottleneck southern Scandinavian samples.

**Table 1:** Indices for genetic diversity of the 41 scandinavian brown bear mitogenomes (11,412 bp).

	*n*	h	S	Hd	π	Tajima’s D
Post-bottleneck clade 1a	10	1	0	0.000	0.00000	-
Pre-bottleneck clade 1a (incl. ancient)	9	9	19	1.000	0.00044	-
Pre-bottleneck clade 1a (excl. ancient)	6	6	14	1.000	0.00047	-0.732 (*p* > 0.10)
Post-bottleneck clade 3a	11	3	11	0.636	0.00033	0.488 (*p* > 0.10)
Pre-bottleneck clade 3a	11	4	12	0.673	0.00034	-0.218 (*p* > 0.10)

Indices are given for the western (clade 1a) and eastern (clade 3a) haplogroups, before and after the bottleneck. Displayed are sample size (n), number of observed haplotypes (h), number of polymorphic/segregating sites (S), haplotype diversity (Hd), nucleotide diversity (π) and Tajima’s D.

## Discussion

In this study, we generated 41 ancient, historical, and modern Scandinavian brown bear mitogenomes and were able to further investigate the Scandinavian brown bears’ recolonisation history and the repercussions of the recent population decline. Our results suggest that Scandinavia was recolonised by several mitochondrial lineages from the south, and a single mitochondrial lineage from the north. Furthermore, we find that the recent human-induced population decline likely resulted in more severe genomic consequences for brown bears in southern compared to northern Scandinavia.

With the retreat of the Fennoscandian ice sheet, it is thought that brown bears recolonised Scandinavia from two directions. This hypothesis was based on analyses of short mitochondrial DNA sequences, where brown bears in southern Scandinavia were shown to carry mitochondrial clade 1a and in northern Scandinavia carry mitochondrial clade 3a. It is also thought that these expanding populations met in central Sweden^24,32^, where a contact zone was formed due to the philopatric behaviour of female brown bears^43,44^. Using mitogenomes, we confirm this mitochondrial phylogeographic pattern (Fig. 2), with the southern Scandinavian brown bears falling into clade 1a and the northern Scandinavian brown bears falling into clade 3a. We did not find any southern brown bears carrying clade 3a among the ancient samples, as reported by Ersmark et al.^30^, or clade 1b which has been reported in Denmark^32^. This absence can likely be due to the limited nature of the dataset. Although speculative, it is possible that clade 3a and clade 1b did not persist in southern Scandinavia after the initial recolonisation, as we do not find any evidence of them in the considerably larger historical and modern datasets either. Moreover, it may indicate that the recolonisation event of Scandinavia did not temporally overlap with the presence of clade 1b in Denmark.

During the Pleistocene/Holocene transition, there were two short periods when the south of Scandinavia was temporarily connected to Denmark and Germany via land bridges. The first one occurred between 13.1 and 12.7 kya, and the more recent one between 12.1 and 10.3 kya (after Björck^45^, Radiocarbon dates calibrated by Herman et al.^20^). Since the inundation of the second land bridge, southern Scandinavia has been disconnected from the central European mainland. Although it is unclear whether brown bears crossed during the first or the second land bridge, it should be noted that indirectly dated fossils suggest that brown bears were present in Denmark around 13.4 kya^46^. This indicates that the ecological flexibility of brown bears^47,48^ allowed them to expand northwards close to the retreating ice edge. Taken together, it is therefore possible, and perhaps likely, that brown bears crossed using the first land bridge and thus was one of the earliest species to recolonise Scandinavia, a speculation shared in previous studies^27,32^. Thus, ~ 13 kya should be considered as the earliest possible recolonisation date.

The number of Scandinavian brown bear mitochondrial lineages with divergence dates preceding 13 kya can thus give insight into how many female lineages were involved in the recolonisation of southern Scandinavia. Our results support a multiple female lineage scenario (Fig. 3). The first divergence among mitochondrial lineages in our Scandinavian dataset was estimated to 23.7 kya (95% HPD: 30.7–17.5 kya), which precedes the earliest possible recolonisation event. The second split was estimated to 18.3 kya (95% HPD: 24.9–12.4 kya), for which the median node age again predates 13.1 kya, but the lower range of the 95% HPD overlaps with the presence of the first land bridge. Hence, our data indicates that the south of Scandinavia was recolonised by at least two, and possibly three mitochondrial lineages. The genetic timelines suggest that the recolonisation of southern Scandinavia was not a straightforward process but possibly involved more complex migration patterns. Two plausible scenarios emerge from our data: (1) Simultaneous recolonisation: Multiple female lineages may have crossed the land bridges simultaneously during one of the available windows, suggesting a migration possibly driven by favourable environmental conditions and resource availability; (2) Sequential recolonisation: Alternatively, the region could have been colonised in phases, with different groups migrating during the separate availability of the two land bridges, indicating a staggered recolonisation process. Interestingly, it seems that during the early Holocene, more mitochondrial lineages were present in southern Scandinavia compared to today. The mitochondrial lineage leading to Sw4 (age: 10,290+-57 calibrated years before present) seems to not have left any descendants in the 19th and 20th century and present-day populations, which likely is a result of recurrent Holocene genetic drift.

In contrast, our analyses suggest that the north of Scandinavia was initially recolonised by a single mitochondrial lineage (Fig. 4), since the most recent common ancestor of the northern Scandinavian brown bears was estimated to 11.4 kya (95% HPD: 15.0–8.1 kya). This timing coincides with the retreat of the Fennoscandian ice sheet^12^. The presence of modern brown bears from Finland within the same clade as Scandinavian brown bears could reflect the northern connectivity. Indeed, recent gene flow between Finnish and Scandinavian male brown bears has been identified^49^. Further research using a temporal approach including ancient, historical, and modern samples from Sweden, Norway and Finland could provide additional insights on this matter.

It is worth acknowledging that this study relies solely on mitochondrial genomes, a single locus genetic marker with a strictly maternal mode of inheritance and no recombination. Therefore, mtDNA can only provide insight into the female population history. It is not surprising, then, that mitochondrial and nuclear genomes may yield discordant results. A recent analysis of modern nuclear genomes suggests that bears from southern and northern Scandinavia are more similar than previously thought^50^. Several factors, including sex-biased gene flow or a single recolonisation followed by diversification, could explain this autosomal pattern. However, given that our modern, historical and ancient mitogenomes are highly consistent with two different re-colonisation routes, we hypothesise that the observed autosomal similarity between bears from southern and northern Scandinavia in de Jong et al.^50^ is likely due to male-mediated gene flow during the Holocene.

Using short mitochondrial sequences, Xenikoudakis et al.^39^ reported a higher pre-bottleneck mitochondrial diversity in southern compared to northern Scandinavia. Our results show that this pattern likely is due to a higher number of founding lineages in southern Scandinavia.

To further investigate the impact of human persecution on the Scandinavian brown bears, we assessed the change in mitochondrial diversity across the past 100 years. We identified a high mitochondrial diversity in Scandinavian brown bears prior to the bottleneck, especially in the southern population where each pre-bottleneck individual had a unique haplotype (Fig. 5; Table 1). This differs from the results of Bray et al.^32^, which reported a low mitochondrial diversity in Scandinavian brown bears already since the early Holocene using short mitochondrial markers.

As a consequence of the bottleneck, all mitochondrial diversity was lost for southern Scandinavian brown bears, with only a single haplotype remaining in the contemporary population. In contrast, the northern Scandinavian brown bears retained most haplotypes (Fig. 5; Table 1). These findings are in line with the results presented in Xenikoudakis et al.^39^, who, based on short mitochondrial markers and microsatellites identified a reduction of mitochondrial haplotypes and allelic richness in Scandinavian brown bears following the bottleneck, which was more pronounced in the south. Moreover, lower genetic diversity in southern Scandinavia today is also in agreement with the findings in de Jong et al.^50^, who reported longer runs of homozygosity in southern Scandinavian brown bears compared to the north^50^. Furthermore, our findings of a severe loss of mitochondrial diversity in southern Scandinavian brown bears matches the expectations for bottlenecked populations. It is in agreement with reports for other species experiencing population declines, such as the Iberian lynx (*Lynx pardinus*)^51^, Western European and Iberian wolves (*Canis lupus*)^52,53^, and Central European moose (*Alces alces*)^54^. Interestingly, however, other large carnivores in Scandinavia, such as the wolverine and wolf, have been shown to have had low mitochondrial diversity already prior to their 20th century bottlenecks^55,56^. This suggests that the Scandinavian brown bear had a larger long-term (pre-bottleneck) effective population size compared to sympatric wolverines and wolves.

Our results suggest that the bottleneck had less impact on the northern Scandinavian brown bears. Moreover, haplotype diversity in the northern post-bottleneck Scandinavian bears is similar to that reported for post-bottleneck bears from Finland, which was speculated to be a result of population recovery due to migratory bears from Russia^57^. For Scandinavian brown bears, however, we suggest a different scenario. The difference in impact of the bottleneck likely mirrors the brown bear’s extermination in large parts of southern Scandinavia, and the continuous presence and larger numbers reported for brown bears in central and northern regions^34,35,38^. The historical pressure has led to today’s distribution of the southern lineages now restricted closer to the contact zone in Jämtland. The only haplotype found among southern modern samples of this study is a continuation of the haplotype found in a historical sample (GX0032) from this area. Thus, the southern Scandinavian brown bears likely suffered from a more severe population decline, while mitochondrial diversity in the north was maintained. A similar pattern has been reported for Scandinavian otters (*Lutra lutra*), in which a pronounced loss of genetic diversity was observed in the southern but not in the northern otters^58^.

## Conclusions

In conclusion, our results contribute significantly to the existing body of knowledge on the genetic diversity of Scandinavian brown bears by focusing on mitochondrial genomes. We have provided insights into the maternal lineage and historical patterns of genetic variation. Our study adds depth to the understanding of how the postglacial recolonisation and recent population pressures have shaped the genetic diversity of brown bears in Scandinavia. However, it is worth noting that mitochondrial genomes only represent one genetic marker and that it only reflects maternal ancestry. To obtain a more nuanced picture of the postglacial history and Holocene changes in gene flow and diversity of the Scandinavian brown bear, a temporal approach using autosomal genome-wide markers is necessary.

## Methods

### Sampling

The dataset consists of 41 ancient, historical and modern samples from Sweden and Norway (Fig. 1; Supplementary Table S1). The three ancient bone and tooth samples were dated to ca. 7600 years before present, 10,290 ± 57 calibrated years before present, and 1916 ± 27 calibrated years before present^30^. The 17 historical bone and skin samples date to between the 1830’s to 1940’s, and the 21 modern muscle tissue samples were collected between 2007 and 2012. All samples were obtained from museum collections or provided by collaborators (Supplementary Table S1). All ancient and historical laboratory work was carried out in facilities dedicated for working with degraded DNA at the Museum of Natural History and the Centre for Palaeogenetics in Stockholm.

### DNA extraction, library build and sequencing

#### Modern samples

The 21 modern samples were collected from muscle tissue and extracted using the DNeasy Blood and Tissue kit (Qiagen, Hilden, Germany), following the manufacturer’s instructions. The DNA extracts were sent to National Genomics Infrastructure (NGI), Stockholm for modern DNA library preparation, using the Illumina TruSeq PCR-free kit (350 bp), and sequenced on 0.5 lanes of a Illumina NovaSeq 6000 S4-300 flow-cell, with a paired-end 2 × 150 bp setup.

#### Historical samples

For the 17 historical samples, DNA extracts from 16 bone samples and one skin sample were obtained from a previous study^39^. Double stranded libraries were built following a modified version of the Meyer Kircher protocol^59^, with additional treatment with 6 units of USER Enzyme mix (New England Biolabs) and AccuPrime™ Pfx DNA Polymerase (Thermo Fisher Scientific Inc.). A unique indexing primer combination was used per library to ensure unique labelling. Five to six amplifications per sample were pooled and cleaned with Agencourt AMPure XP beads (Beckman Coulter, Brea, CA, USA). Each cleaned pool was run on a high-sensitivity DNA chip on a Bioanalyzer 2100 (Agilent, Santa Clara, CA, USA) to assess DNA concentration and fragment size distribution. All historical samples were pooled in equimolar ratios and sequenced on one lane of an Illumina HiSeq 2500 platform with a paired end 2 × 125 bp setup.

#### Ancient samples

DNA extracts were obtained from a previous study^30^. Following^60^, double stranded Illumina libraries were built with a modified version of the Meyer Kircher protocol^59^, using 3 Units of USER enzyme. DNA library products were purified with Agencourt AMPure XP beads (Beckman Coulter, Brea, CA, USA). DNA concentration and fragment size distribution was measured on a high-sensitivity DNA chip on a Bioanalyzer 2100 (Agilent, Santa Clara, CA, USA). One index PCR for Sw1, Sw4 and Sw9 were initially sequenced on a Illumina Novaseq 6000 S4-200 v1.5 platform, with a paired-end 2 × 100 bp setup. Sw1 and Sw4 were sequenced in a second sequencing run with the same settings, to further increase coverage. For Sw9 sufficient yield was reached from the initial sequencing run.

### Data processing

For historical and modern samples, a beta-version of the GenErode pipeline was used, while ancient samples were processed using GenErode v0.4.2^61^. GenErode is a bioinformatics pipeline to process and analyse ancient, historical, and modern genomic data^61^. Adapter trimming and read merging was done for modern samples using TrimGalore v0.6.5 (https://github.com/FelixKrueger/TrimGalore), for historical samples using a modified version of SeqPrep v1.1 (https://github.com/jstjohn/SeqPrep) as in^62^, and for ancient samples using fastp v0.22.2^63^. Reads shorter than 30 bp were discarded. In order to avoid nuclear mitochondrial DNA segments (NUMTs), the trimmed and merged reads were mapped to the nuclear-mitochondrial polar bear (*Ursus maritimus*) reference genome [NCBI RefSeq accession no. GCF_017311325.1;^64^]. For mapping sequenced DNA reads, the BWA v0.7.17 mem algorithm^65^ was used for modern samples, and the aln algorithm^66^ with specific ancient DNA settings (deactivated seeding: -l 16500, more substitutions allowed: -n 0.01, up to two gaps allowed: -o 2) was used for historical and ancient samples^62^. Duplicates were marked with Picard v2.22.4 MarkDuplicates (https://broadinstitute.github.io/picard/) for modern and removed with SAMtools v1.9^67^ for historical and ancient samples, and IndelRealigner from GATK v3.7^68^ was used to realign reads around indels. A second round of duplicate removal using the samremovedup.py script from GenErode v0.4.2 was performed for Sw1 and Sw4, to remove remaining duplicates after merging of bam files of the same samples but different index PCRs. For Sw9 and the historical and modern samples, only one index per sample was used, thus no second round of duplicate removal was required. Mitochondrial genomes of all samples were extracted using samtools v1.9 with filtering for mapping quality (-q30), and consensus sequences were called with ANGSD v0.933^69^ using the majority rule and filtering for quality score (-minQ 30), mapping quality (-minmapQ 30), as well as minimum coverage of 3X per site, with ambiguous sites being called as N.

### Computational analyses

#### Alignment

We aligned the 41 Scandinavian brown bear mitochondrial genomes in MEGA11^70^. Following Fortes et al.^41^, we excluded a section of the D loop. For phylogenetic and divergence time analyses, we furthermore excluded the Leu1, ND1, Ile, Gln and Met partitions since they consisted of unknown sites (N) only. We then aligned our 41 mitogenomes with additional 408 published mitogenomes, resulting in a 15,118 bp alignment of 449 brown and polar bears (Supplementary Table S4).

#### Phylogenetic relationship and divergence

A maximum clade credibility tree of 449 brown and polar bear mitogenomes was generated in BEAST v1.10.4^71^, using a strict molecular clock, a previously estimated substitution rate of 2.48 × 10^−8^ mutations per site per year^8^, and a constant size coalescent tree model. HKY with a gamma distribution and invariant sites was selected as the nucleotide substitution model based on the Bayesian Information Criterion in Jmodeltest v2.1.10^72,73^. Tip dates were specified as radiocarbon dates, previously published molecular date estimates, or estimated ages based on archaeological context (Supplementary Tables S1, S4 ). The age of Sw1 was estimated by sample tip dating, using a uniform prior with a mean of 7600 years ago and a range of 0 to 50,000 years ago. We ran 100 million MCMC iterations, sampling every 5000 states. The BEAST output was verified for convergence and effective sample size of the chains in Tracer v1.7.2^74^, and a consensus tree (with 10% burn-in removed) was generated in TreeAnnotator v1.10.4 obtained from the BEAST toolkit. The maximum clade credibility phylogenetic tree was visualised in Figtree v1.4.4 (https://github.com/rambaut/figtree/releases/tag/v1.4.4) and Linearity Curve v.5.0.0 (https://www.linearity.io/curve/). See Supplementary Figure S1 for all individual samples.

#### Mitogenome networks

To avoid biases, we excluded ambiguous sites (N) which resulted in a 11,412 bp alignment of the 41 Scandinavian brown bears. We generated a temporal haplotype network of the 41 full mitochondrial genomes using the R script TempNet v1.8^75^. To enhance clarity, the haplotype network was subsequently redrawn using Linearity Curve v.5.0.0. Additionally, a median-joining haplotype network was generated in PopART with default parameters and epsilon = 0^76^ (Supplementary Figure S2).

#### Mitogenome diversity

For mitochondrial genetic diversity analysis, we used the 11,412 bp alignment of the 41 Scandinavian brown bears, excluding unknown sites (N). We calculated mitochondrial genetic diversity indices including haplotype diversity (Hd), nucleotide diversity (π) and Tajima’s D^77^ using DNAsp v.6.12.03^78^. Tajima’s D was calculated excluding the three ancient samples. For this analysis, the dataset was split by haplogroup and time periods; pre-bottleneck clade 1a (*n* = 9), post-bottleneck clade 1a (*n* = 10), pre-bottleneck clade 3a (*n* = 11), post-bottleneck clade 3a (*n* = 11) (Supplementary Table S1).

## Electronic supplementary material

Below is the link to the electronic supplementary material.

Supplementary Material 1

Supplementary Material 2

## Data Availability

Mitochondrial sequences obtained in this study are deposited in GenBank (accession numbers PQ334008-PQ334048). The xml-file used as input for the BEAST analysis is available at the Zenodo repository (DOI: 10.5281/zenodo.13744721).
