# Supplementary material for: Heterochronous mitogenomes shed light on the Holocene history of the Scandinavian brown bear
Source: Sci Rep. 2024 Oct 22;14:24917. doi: 10.1038/s41598-024-75028-6 (PMC11496541; doi:10.1038/s41598-024-75028-6)
Supplement: Supplementary file 2 — Supplementary Material 2 [file 41598_2024_75028_MOESM2_ESM.pdf]

Supplementary Information 2

Heterochronous mitogenomes shed light on the Holocene history of the Scandinavian brown bear

Isabelle Sofie Feinauer[1-3*], Edana Lord[1,2], Johanna von Seth[1-3], Georgios Xenikoudakis[1,4], Erik Ersmark[1], Love Dalén[1-3], Ioana-Nicoleta Meleg[1,3,5*]

Affiliations:

1) Centre for Palaeogenetics, Svante Arrhenius väg 20C, Stockholm 106 91, Sweden
2) Department of Zoology, Svante Arrhenius väg 18C, Stockholm University, Stockholm 106 91, Sweden
3) Department of Bioinformatics and Genetics, Swedish Museum of Natural History, Box 50007, Stockholm 104 05, Sweden
4) Department of Archaeology and Ancient Culture, Wallenberglaboratoriet, Lilla Frescativägen 7, Stockholm University, Stockholm 106 91, Sweden
5) Emil G. Racoviță Institute, Babeș-Bolyai University, Clinicilor 5-7, 400006 Cluj-Napoca, Romania

*Corresponding authors (isabelle.feinauer@su.se, ioana.meleg@ubbcluj.ro)

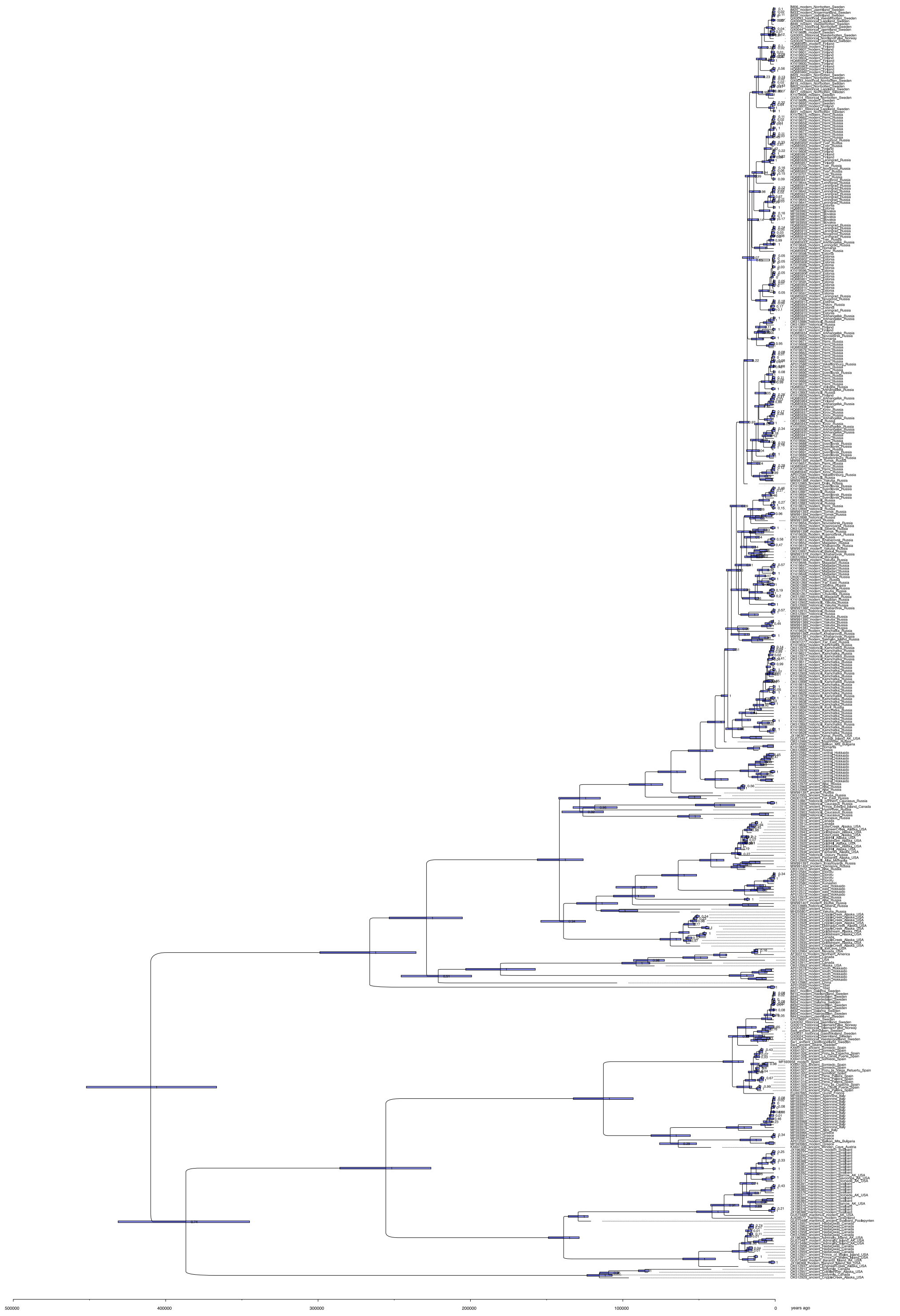

Supplementary Figure S1: Time-calibrated phylogeny based on the 15,118 bp alignment of 449 brown and polar bear mitogenomes generated in BEAST. 2.48 x 10-8 substitutions per site per year was used as the substitution rate. The y-axis is given in years ago. Labelling follows SampleID or Genbank Accession Number, time period, and country. Branch posterior probabilities are displayed, while bars indicate the 95% highest posterior density (HPD) for nodes.

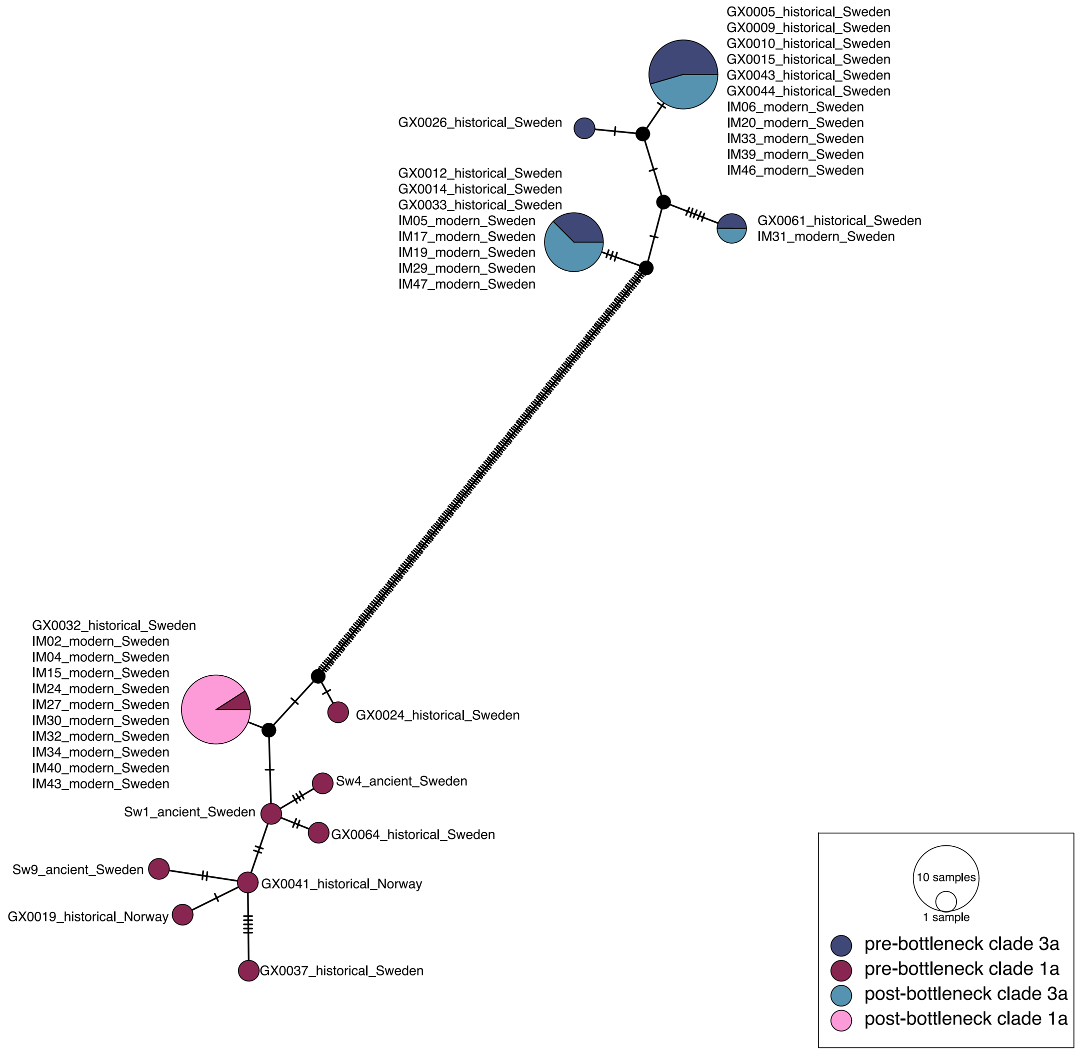

Supplementary Figure S2: Median-joining haplotype network for the 41 brown bear mitogenomes (11,412 bp), generated in PopART. Coloured circles represent the Eastern and Western clades, separated by time period, pre- and post-bottleneck, as indicated in the legend. Black dashed represent segregation between haplotypes.
